# Objective monitoring of Insecticide-treated bednet use to improve malaria prevention: SmartNet development and validation

**DOI:** 10.1371/journal.pone.0168116

**Published:** 2017-02-03

**Authors:** Paul J. Krezanoski, Jeffrey I. Campbell, Data Santorino, David R. Bangsberg

**Affiliations:** 1 Department of Medicine, Zuckerberg San Francisco General Hospital, University of California, San Francisco, San Francisco, California, United States of America; 2 Department of Pediatrics, Zuckerberg San Francisco General Hospital, University of California, San Francisco, San Francisco, California, United States of America; 3 Harvard Medical School, Boston, Massachusetts, United States of America; 4 Mbarara University of Science and Technology, Mbarara, Uganda; 5 Oregon Health Sciences University-Portland State University School of Public Health, Portland, Oregon, United States of America; University of Crete, GREECE

## Abstract

Malaria is a serious health concern for three billion people worldwide, killing nearly 600,000 people a year. Insecticide-treated bednets (ITNs) are an effective and valuable tool for preventing malaria and hundreds of millions of ITNs have been distributed throughout sub-Saharan Africa. Nevertheless, our current methods for measuring ITN use are inadequate to inform malaria prevention programs. The most common method, self-reported ITN use, is limited by 1) social desirability, 2) recall and 3) sampling bias. An acceptable objective and longitudinal method of assessing adherence to ITN use would improve our ability to better understand the determinants of ITN use and design more effective malaria prevention interventions. We describe the development and initial proof-of-concept validity testing of an ITN adherence monitoring tool called SmartNet. SmartNet uses conductive thread interwoven into an ITN and a microcontroller to detect the state of the ITN. We tested SmartNet among five volunteers using the device over their beds in Boston, USA for two weeks with the goal of evaluating device reliability, accuracy and acceptability to inform future device improvements. The device recorded data for 63.1% (35172/55711) of installed two-minute time intervals, with 97.3% (19990/20539) of the recording errors relating to battery failures. Overall, the device was 71.7% (25204/35172) accurate in determining the state of the ITN (whether it was folded up or unfurled) and performed significantly better at detecting an unfurled ITN than a folded ITN, 77.3% versus 68.4% (p<0.001). Participants noted no significant acceptability concerns and all participants felt SmartNet was easy or very easy to use. SmartNet is a novel approach to objectively measure ITN adherence over time. Our results suggest a variety of device improvements to both extend reliability and improve performance of SmartNet prior to deployment in a malaria-endemic setting.

## Introduction

Malaria kills nearly 600,000 people per year world-wide [1]. Insecticide-treated bednets (ITNs) are highly effective for malaria prevention [2]. Household ownership of an ITN results in an 18–23% reduction in all-cause child mortality, on par with the effect of the measles vaccination in reducing mortality in children under 5 years of age [3]. ITNs are widely available and are a crucial component of the world’s malaria control strategy. The World Health Organization (WHO) recommends that all three billion people at risk of malaria utilize bednets for protection. In 2014 alone, 214 million ITNs were distributed worldwide [1].

Improving ITN effectiveness is limited by an incomplete understanding of factors related to ITN adherence. This is due, in part, to methodological limitations in measuring ITN use. The most commonly used measure of ITN adherence is self-reported use of an ITN during the prior night [4,5,6,7,8]. However, self-reported ITN adherence suffers from potential biases that make it an unreliable measure of ITN use [8], including a) social desirability bias, b) recall bias and c) sampling bias related to queries focused on a single night which may miss important seasonal and other temporal variations in use [5].

Objective measures of ITN use have been used to overcome limitations in self-report, but each measure has its own logistical and methodological challenges. Surprise night visits, for example, are arguably the most objective adherence measure [9,10,11], but are logistically challenging and poorly accepted due to privacy concerns. Daytime visual confirmation during observer home visits can confirm that an ITN is installed over a bed [12,13], but installation alone does not ensure nightly use.

While electronic monitoring is commonly used to measure medication adherence, including in sub-Saharan Africa settings [14,15], there are few approaches to electronic ITN adherence monitoring [16]. Objective electronic ITN adherence monitoring would improve our ability to characterize patterns of ITN use and determinants of ITN adherence. This improved understanding could inform the development of theoretical behavioral models of ITN adherence in order to improve ITN effectiveness in households at risk of malaria. We describe the development and initial proof-of-concept validity testing of an ITN adherence monitoring tool called SmartNet among volunteer users using SmartNet over their beds in Boston, MA, USA.

## Methods

### Recruitment

Five medical students were recruited through emails and fliers at a medical school in Boston, MA. Participants were enrolled in the order that they replied to advertisements and pending availability for the installation of a SmartNet in their home. Eligibility included having a twin or queen size mattress, age >18 years and willingness to keep a written log of the dates and times of use of SmartNet use over a two week period.

### SmartNet development

In 2011, we developed the battery powered SmartNet device to electronically collect and store an objective and longitudinal record of whether an ITN is folded up or unfurled over bed in malaria endemic settings. SmartNet was designed to be integrated into an WHO-approved ITN and intended to be installed and used like a regular ITN in households. Typical ITNs are composed of netting impregnated with insecticide. The ITNs provide both a physical barrier and a chemical insecticide that kills mosquitoes that come into contact with the net. ITNs are hung from a ceiling or walls and are usually rectangular (~5x6 feet) with a top and four sides that fall along the edges of a bed to provide protection from mosquitoes. ITNs are unfurled during sleeping hours and, when not in use, are typically folded up or knotted.

The SmartNet device is made up of two main components: 1) a mechanism for detecting whether the ITN is folded up or unfurled and 2) a microcontroller to classify and store the SmartNet detected result on a memory card every two minutes. SmartNet uses conductive thread sewn into a rectangular ITN to form three electrical circuits that detect whether the ITN is folded or unfolded. To accomplish this, the microcontroller sends a small current through the conductive thread using insulated wires. When the ITN is folded or knotted for storage, a circuit is completed between two or all of three electrical circuits described above implying that the SmartNet is “folded up”. Alternatively, if none of the circuits is completed, the SmartNet is classified as “unfurled”.

The ITN used for this study was a WHO-approved twin bed-sized (4x6x7 foot) NetMark net purchased in Mbarara, Uganda in 2013. The conductive thread is a stainless steel conductive thread from Adafruit (Product ID: 640) with a resistivity of 16 ohms. The 10 microAmp current utilized by SmartNet is below human detection and orders of magnitude below the 60 milliAmps necessary to induce pain or other harm [17]. A Brother HC1850 sewing machine was used to sew the conductive thread into the ITN in the three distinct circuits using a set pattern. The sewing pattern was modeled using freeCAD (version 0.12) and was designed to increase the likelihood of circuit connectivity when the ITN was folded up or tied into a knot. This design was based on repeated laboratory observations of a table-top miniaturized ITN replica.

The microcontroller was an Arduino Uno with a customized SmartNet shield. The SmartNet shield has a switch for power on and off, buttons for test and reset functions, a micro SD memory card, button cell battery to maintain the system clock, lithium ion battery plug and screw-top input ports for the three conductive thread circuits. The Arduino was programmed with a hibernation mode to save power between two minute measurement intervals. To further extend battery life, the net state is stored locally on the microcontroller memory and only writes to the external memory every after 16 readings (32 minutes) to reduce the power required to boot up the SD memory card. A lithium ion battery was used to externally power the microcontroller and shield.

### Study protocol

All participants used the same SmartNet device. At the installation visit, informed consent was obtained and then a baseline survey was performed to obtain basic demographic data and previous experience with ITNs. Next, a SmartNet was mounted over the participant’s bed using portable microphone stands with 10 pound weights at the bases to provide stability. SmartNets were hung by attaching the four loops at the corners of the ITN to the extended portable microphone stands placed at the four bed corners. SmartNet was installed on these microphone stands to avoid incurring damage to participant walls or ceilings using nails to hang the nets.

SmartNet functionality was confirmed upon installation using a “test mode” function on the device. The test function provides visual feedback depending on the state of the ITN. For example, an ITN which is unfurled (i.e. there are no connections between conductive circuits) displays a simple blink of three LED lights and then turns off. However, when an ITN is folded up (i.e. at least two circuits are in contact) the LEDs light up for 5 seconds corresponding to the circuits that have come in contact. At installation and at all home visits the SmartNet was confirmed to be working properly using a standardized protocol based on the results of this test function. The protocol involved testing the SmartNet when unfurled and then folded up in five different ways (once with all sides gathered together and tied into a knot, and then four times with the device folded up from each of the four sides of the net).

Following mounting and confirming that the SmartNet was functioning correctly, participants were provided with a blank diary to record the date, time and state of the SmartNet (UP or DOWN). Participants were requested to change the ITN state as frequently as possible to maximize data collection (e.g. whenever they noticed the net) and then note the change in the diary.

At the midpoint follow-up visit (after one week), SmartNet data was downloaded from the memory card, use diaries were collected and device functionality and battery function were confirmed using the test protocol described above. If a SmartNet was not functioning, the battery was replaced. If battery replacement did not restore function, the microcontroller and net were replaced. Participants were interviewed on their impressions of acceptability of SmartNet (i.e. perceived ease of use, impact on sleep, concerns about monitoring) using a combination of structured and open-ended questions. During the final visit (after two weeks), the acceptability interview was repeated and the SmartNet, memory card and use diaries were collected.

### Study endpoints

The primary objectives for this proof-of-concept study were to measure 1) SmartNet reliability, defined as the proportion of total installed time in which SmartNet provided ITN state data, and 2) the performance of SmartNet in accurately determining the state of the ITN relative to the participant-generated use diaries. The secondary objectives were to evaluate the acceptability of SmartNet and identify device malfunctions and acceptability elements for design modifications.

### Statistical analyses

The time and date of each change in the ITN state (UP→DOWN or DOWN→UP) was extracted from the diaries and used as the referent measure to determine SmartNet performance characteristics.

The reliability of SmartNet was defined as the proportion of two minute intervals that the SmartNet recorded time-stamped data on the memory card. The performance of SmartNet was assessed according to its percent accuracy in correctly classifying the state of the ITN relative to the use diaries. A receiver operating curve (ROC) was constructed based on the sensitivity and specificity of the SmartNet data in relation to the ideal log (i.e. use diary) according to projected two minute intervals (Table 1).

**Table 1:** Accuracy of SmartNet in determining ITN state.

		ITN state according to participant diaries	
		Folded up	Unfurled
SmartNet-detected	Folded up	True Positive: ITN folded up and detected by SmartNet	False Positive:ITN unfurled but SmartNet indicating folded up
	Unfurled	False Negative:ITN folded up but SmartNet indicates unfurled	True Negative: ITN unfurled and SmartNet indicates unfurled

Sensitivity = True Positives / (True Positives + False Negatives) = Accuracy of SmartNet in detecting a folded up net

Specificity = True Negatives/ (True Negatives + False Positives) = Accuracy of SmartNet in detecting an unfurled net

SmartNet acceptability data were analyzed as a summary of salient themes from open-ended questions about user impressions and experiences with the device (Table 2). Device malfunctions were recorded, along with what is known about the circumstances surrounding the malfunction.

**Table 2:** Questions about perceptions of SmartNet and experience with use.

Device Acceptability
How easy was it to use the SmartNet?
Does the SmartNet interfere with regular use of the bednet?
Are there particular things you like/do not like about the SmartNet?
Objective Monitoring
How concerned are you that your use of the bednet is being monitored by the SmartNet?
Is there a way that the monitoring of your bednet could bother you less?

All data were collected on paper forms and entered manually into Microsoft Excel 2007 (Microsoft Corporation, Redmond, WA). Quantitative data were analyzed in Stata 10 (Statacorp, College Rd, TX).

### Ethical review

The study was approved by the Partners Human Research Committee Institutional Review Board. All participants provided written informed consent.

## Results

All five participants had SmartNets successfully installed in their homes, completed baseline demographic and subsequent acceptability surveys, and filled out valid SmartNet use diaries over the study period. The participants had a mean age of 27.2 (range: 25–30) and three of five (60%) were female. They were all graduate medical students. One participant had never used an ITN previously. The other four had used ITNs in Latin America (two participants) and Africa (two). Duration of experience with ITNs ranged from a few weeks (one) to a few months (two) to a few years (one).

### Device reliability

SmartNet devices were installed for a total of 77 days in participant households comprising a total of 55,711 possible two minute intervals of installation time (Table 3). Participant 1 used SmartNet for 21 days instead of the scheduled 14 days due to a delay in device retrieval. Overall, the device provided a decipherable recording of the SmartNet state for 63.1% (35172/55711) of possible two minute intervals.

**Table 3:** Total two minute intervals installed and captured by SmartNet.

Participant	Dates installed	Intervals recorded/installed
1	February 9—March 2, 2014	97.6% (14740/15106)
2	March 16—March 29,2014	44.7% (4290/9593)
3	April 7—April 21, 2014	0% (0/10693)
4	May 23—June 6, 2014	98.2% (9950/10133)
5	June 6—June 20, 2014	60.8% (6192/10186)
	Total:	63.1% (35172/55711)

There was a total of 20,539 missed measurements which were due either to battery failures (97.3%, 19990/20539) or uncategorized error (2.7%, 549/20539). First, 77.9% (15996/20539) of failed measures were attributable to a loss of time stamps caused by a failure of the clock battery (button-cell) on the SmartNet device during use by participant 2 and 3. The SmartNet device produced data for participants 2 and 3, but a large proportion could not be deciphered because of the inaccurate time stamps. Second, failure of the overall power source accounted for 19.4% (3994/20539) of measurement errors. This was most evident during recording of participant 5, where only 10 of 14 days were recorded. Finally, 549 measurement errors (2.7%) occurred even when there was adequate battery power (indicated by recorded measures both before and after the error). These were presumably due to errors in buffering data on the Arduino microcontroller or writing errors on the memory card. Errors due to battery failure were censored from the accuracy analysis, but the latter, uncategorized errors, were retained.

### Device accuracy

**Fig 1:**
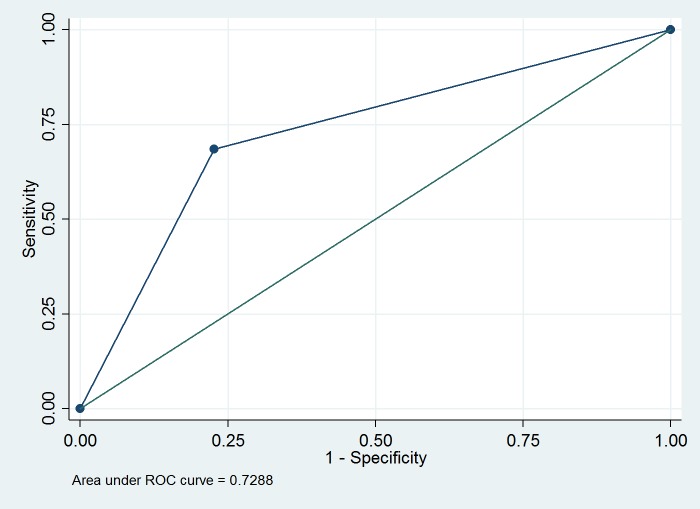
ROC curve for SmartNet detection of bednet state using participant diaries as reference.

Utilizing participant use diaries as a reference measure, SmartNet correctly classified the ITN state for 71.7% (25204/35172) of two minute intervals (Table 4 and S1 File). SmartNet was significantly less likely to correctly classify ITN state when it was folded up than when it was unfurled: 68.4% (95% confidence interval (CI): 67.8%–69.0%) versus 77.3% (95% CI: 76.6%–78.1%) (p<0.001). An ROC curve was generated with an area under the curve (AUC) of 0.729 (95% CI: 0.724–0.734) (Fig 1). SmartNet was 68.4% sensitive and 77.3% specific in detecting the state of the bednet (i.e. whether the net was up or down) using the participant diaries as reference.

**Table 4:** 2x2 table of SmartNet versus diary ITN state.

		ITN state according to participant diaries		
		Folded up	Unfurled	Total
SmartNet-detected	Folded up	9,854	2,888	12,742
	Unfurled	7,080	15,350	22,430
	Total	16,934	18,238	35,172

### Device acceptability

Participants did not identify any features of SmartNet use that caused particular inconvenience. Two participants noted that the ITN was too small for their beds. Three participants noted that the diary entries were inconvenient. Two participants also described occasional toppling of the microphone stands used to support the ITNs over their beds. Overall, every participant rated the SmartNet device as easy or very easy to use and none of the participants were concerned about the fact that their ITN use was being objectively monitored.

## Discussion

SmartNet is a novel tool for assessing the use of ITNs. SmartNet utilizes conductive thread integrated into an ITN to act as a measure of the state of a net, i.e. whether the net is folded up for storage or unfurled and in use. This approach holds promise for allowing objective and longitudinal measurement of ITN use in malaria-endemic settings.

SmartNet provides two main advantages over current methods for determining ITN use. First, the objective nature of the SmartNet measurement avoids the bias inherent in self-reports of ITN use. This objectivity could greatly improve the accuracy of ITN use assessments in surveillance studies, with the potential for altering current malaria prevention strategies related to ITNs. Secondly, SmartNet can provide finer temporal resolution in measurements of ITN use than currently available methods, resulting in a complete longitudinal record of the use-state of an ITN over time. This is a significant advance over current surveillance methods which rely on one-time measurements at household surveys, usually asking about use of the ITN the night before.

SmartNet was designed to be as inconspicuous as possible. It is integrated into an existing ITN using simple techniques, a sewing machine and conductive thread. The microcontroller used in the study reported here is about the size of a wallet, but future iterations should result in smaller components. With future iterations, it should be possible to make the entire SmartNet system even more inconspicuous and thus a more ideal adherence monitoring tool. The lower bound on the size of a SmartNet microcontroller would likely be limited by the size of a miniature memory card (~1.5 cms).

Proof-of-concept testing at early stages of technology development can provide valuable information for future device improvement. The objective of this study was to determine SmartNet reliability, performance characteristics, acceptability and common malfunctions for design improvements.

SmartNet reliability was compromised by three distinct factors. First, although SmartNet was online and producing data for 92.8% (51717/55711) of all possible two minute intervals, some of these measurements were undecipherable due to an unexpected failure of an onboard button-cell battery used to maintain the system clock for the device. The system clock is required to generate time stamps for the SmartNet data. Further investigation indicated that the failure of the button-cell battery was due to the custom shields inadvertently leaching power from the button-cell batteries even in power saving modes. Redesign of the circuit board to avoid this energy drain or possibly simply replacing the button-cell battery before use could prevent premature battery drainage. Second, there was unexplained variability in battery performance. We discovered that one particular lithium-ion battery and certain devices themselves performed differently. This will require explicit power draw-down tests of devices before deployment. We are considering using a less expensive and more consistent power source (AA batteries) to extend battery life and independent device function for weeks to months at a time. A final reliability issue was the lack of recorded data even when battery power seemed to be working adequately. This accounted for only a small fraction of the overall failures (2.7%, or 549 of 55,711 potential observations), but will require future exploration to differentiate between a software (related to the buffering system used to save power) or hardware issue (related to the memory cards).

SmartNet was 71.7% accurate in classifying the state of the ITN. The performance characteristics of the SmartNet device were, in practice, poorer than we had anticipated from lab tests. One issue was the problem detecting ITNs in a folded up position. There are a variety of possible modifications to address this poor sensitivity in detecting a folded up net. First, we could alter the sewing patterns for the conductive thread to include more conductive thread. This would increase the surface area for potential contact between the top and side circuits. This presents an optimization problem, because there is a theoretical maximum of making the *entire* net conductive, thus maximizing sensitivity. Increasing conductive thread makes the thread more conspicuous, increases cost of materials and labor. A second potential modification is to use conductive fabric strips instead of conductive thread. These conductive strips are less likely to be lost within the netting and thus are more likely to make contact with each other. A third potential modification to improve performance characteristics may be to create an algorithm for removing the noise in the data produced by SmartNet. Due to the occasionally fickle connections between circuits, there are often isolated instances or short runs of incorrect classifications in between large groups of correct classifications. An algorithm which detects and scrubs data to remove incorrect classifications could improve performance.

In terms of acceptability, the limitations identified in this study are addressable. First, the inconvenience noted due to the small size of the ITNs can be managed with utilizing larger ITNs in future field studies. Second, the toppling of the setup was a side effect of the use of microphone stands to support the hanging SmartNet. This will not be an issue in malaria-endemic settings where ITNs are routinely secured using nails and string to house walls. Finally, the inconvenience of the requirement to fill out the use diary is, again, inherent to the study situation; we do not anticipate using diaries in future studies. Preliminary studies we have performed in Uganda suggest that the appearance and concept of SmartNet is acceptable to most women there [18].

The main limitation of the current study is that it was performed among a small number of volunteers living in a non-malaria endemic setting over a limited, two week, interval. The device may perform differently in the environmental conditions present in households in malaria-endemic settings. Furthermore, medical students in Boston are likely to interact with the technology differently than households in malaria endemic settings. Nevertheless, our participants found SmartNet to be acceptable and challenges were mostly related to the requirement for diary keeping and the inconvenience of using an ITN itself, rather than the monitoring strategy. SmartNet detects whether or not an ITN is folded. It is possible that inappropriate use of ITNs (such as for chicken coops or fishing) could result in data that appears to mimic use, though confirmation of the location of the device during household visits would likely help detect this problem. Finally, because the participants may not have accurately recorded all furling/unfurling transitions in the diary which was used as the referent measure, the device may have performed better than suggested by the study results.

The next step for SmartNet includes testing the device in a malaria-endemic setting among actual users of ITNs. Another next step for SmartNet will be a study which obtains multiple different measures of ITN use for comparison and validation, including self-reported use, objective measures (e.g. unannounced night visits) and SmartNet data.

## Conclusion

SmartNet is a novel approach to objectively measure ITN adherence over time. Our results suggest a variety of device improvements related to improving battery performance and increasing conductive material to both extend reliability and improve sensitivity prior to deployment in a malaria-endemic setting.

## Supporting information

S1 File Data files from SmartNet device and participant diaries. (CSV)
